# 725. Complete Blood Count Values Vary in Degree of Change with Day of Fever in Children with Dengue Fever

**DOI:** 10.1093/ofid/ofab466.922

**Published:** 2021-12-04

**Authors:** Melissa E Day, Miguel E Mejia Sang, Yonairy Collado Puello, Elvira J Diaz Brockmans, Stephanie Rivera Defillo, Karla M Taveras Cruz, Javier O Santiago Perez, Maria F Diaz Soto, Rafael Mena, Cesar Mota, Javier Gonzalez del Rey, Carlos Prada, Elizabeth P Schlaudecker, Lisa J Martin, Brittany Simpson

**Affiliations:** 1 Cincinnati Children's Hospital Medical Center, Cincinnati, Ohio; 2 Tecnológico de Santo Domingo, School of Medicine, Santo, Santo Domingo, Distrito Nacional, Dominican Republic; 3 Tecnológico de Santo Domingo, School of Medicine, Santo Domingo, Distrito Nacional, Dominican Republic; 4 Universidad Iberamericana (UNIBE), School of Medicine, Santo Domingo, Distrito Nacional, Dominican Republic; 5 Centro de Obstetricia y Ginecología, Hospital Infantil Dr. Robert Reid, Santo Domingo, Distrito Nacional, Dominican Republic; 6 Pediatric Emergency Department, Hospital Infantil Dr. Robert Reid, Santo Domingo, Distrito Nacional, Dominican Republic; 7 Cincinnati Children's Hospital Medical Center, University of Cincinnati College of Medicine, Cincinnati, OH

## Abstract

**Background:**

Dengue fever (DF) is an acute viral disease which can lead to severe illness, including dengue hemorrhagic fever, marked by thrombocytopenia and hemolytic anemia, as well as end-organ damage. Despite the well-known presentation and prevalence, changes in hematologic markers across the DF course have not been well-described in children. We sought to investigate the association of clinical laboratory values over time with dengue disease progression and outcome in a pediatric population in the Dominican Republic.

**Methods:**

Pediatric participants were enrolled at Hospital Infantil Dr. Robert Reid Cabral in Santo Domingo, Dominican Republic, in a prospective, observational case-based study. Laboratory values, including complete blood count (CBC) indices and dengue titer results, were collected over the course of hospital stay. Using linear mixed models, we assessed whether 13 different CBC values and time trajectories differed by dengue status, including age and sex as covariates. To account for multiple testing, p≤0.0033 was considered significant.

**Results:**

A total of 575 children ages 0 to 211 months met inclusion criteria; 51.8% (n=298) were male, and the median (IQR) age was 59 (14-93) months. Eighty-two percent (n=472) of participants had DF. CBC values across days 1 to 10 of fever in those with and without DF are depicted in Figure 1. Those with DF showed levels dropping more quickly across days of fever for hematocrit and hemoglobin (p≤ 0.002), with a more rapid decline in those with severe DF (p < 0.0001). Those with DF had levels increasing more quickly for mean corpuscular hemoglobin concentration (MCHC), monocyte number, and white blood cell counts (p ≤ 0.003), with those with severe DF having a more rapid increase (p < 0.001). The direction of the change across time differed by DF status for mean corpuscular volume and red blood cell distribution width (RDW) (p ≤ 0.0003), with those with severe DF showing an increase in RDW across day of fever (p= 0.0004).

Figure 1. CBC values across day of fever in dengue (blue) and non-dengue (purple) patients.

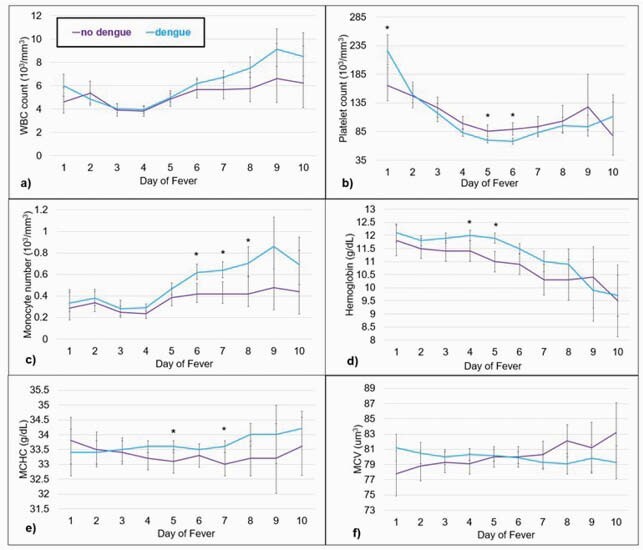

The graph above depicts the following CBC values across day of fever in dengue (blue) and non-dengue (purple) patients: a) white blood cell (WBC) count, b) platelet count, c) monocyte number, d) hemoglobin, e) mean corpuscular hemoglobin concentration (MCHC), and f) mean corpuscular volume (MCV). Values with an asterisk (*) represent significant values (p < 0.0033).

**Conclusion:**

The trajectory of CBC measures differs between those with and without DF, despite similar clinical presentations. These laboratory differences may facilitate a better understanding of the clinical course of DF and may aid in earlier identification of DF in resource-limited settings.

**Disclosures:**

**Elizabeth P. Schlaudecker, MD, MPH**, **Pfizer** (Grant/Research Support)**Sanofi Pasteur** (Advisor or Review Panel member)

